# Supplementary material for: Plasma proteomics identify biomarkers predicting Parkinson’s disease up to 7 years before symptom onset
Source: Nat Commun. 2024 Jun 18;15:4759. doi: 10.1038/s41467-024-48961-3 (PMC11189460; doi:10.1038/s41467-024-48961-3)
Supplement: Supplementary file 1 — Supplementary Information [file 41467_2024_48961_MOESM1_ESM.pdf]

Supplementary Methods 1. ***Protein expression similarities and differences between plasma, serum and CSF***

We determined the expression of the blood-derived biomarkers in our panel and compared them to those in CSF to ascertain if they could have originated from the brain. Additionally, to determine if serum and plasma gave identical results, **phases 0** and **I** were also carried out in plasma samples, but for **phase II** analyses, only serum samples were available. We analysed our panel in paired plasma, serum, and CSF from subjects with various neurological disorders. The analysis revealed large differences between the blood- and CSF-protein expressions in a PCA (results can be found in **Supplementary Figure 4**). An OPLS-DA model comparing CSF and plasma/serum was strongly significant (ANOVA p = 2.0E-20, permutations p << 0.001) and demonstrated that SOD3, PTGDS, CST3, DKK3, FABP5, PRG1, PLD3, APOE and HPX were elevated in CSF while all other proteins, apart from HSP1L, HSPA5 and VCAM1, were higher in the blood-based plasma and serum samples. CSF demonstrated limited correlations with both plasma and serum for all proteins in the predictive panel, apart from MASP2 which was positively correlated (plasma/serum vs CSF, rho = 0.66/0.81). The blood-derived proteins from our panel demonstrated higher values than the CSF proteins for all but DKK3 and PTGDS, which were roughly 80 and 340 times higher, respectively, than the proteins measured in blood.

Individual OPLS-DA models of plasma and serum versus CSF did not differ significantly but reflected the observations from the overall blood- versus brain-based model. Comparing plasma and serum, we found that the levels of the proteins FGA, PGK1, SERPINF2, HSPA5, TUBA4A, GRN, HSPA1L and ADIPOQ differed– all of which were higher in plasma, except for ADIPOQ which was higher in serum.

Investigating the expression of the proteins in our predictive SVM panel in detail, we correlated the paired samples from the three matrices with each other. We noted that plasma and serum demonstrated positive correlations (Spearman's rho 0.75 – 0.93) for the five of the eight proteins: C3, GRN, ICAM1, MASP2 and SERPING1, but not for HSPA5, DKK3 and PTGDS, see **Supplementary Figure 4**. Although a high degree of correlation was observed between plasma and serum, we consider that the performs better in plasma as this is the sample matrix it was developed in.

Supplementary Methods 2. ***Age and sex influence on the protein expression***

In our protein panel, several proteins correlated with age or sex. Elevated levels of CST3, PTGDS, VCAM1, A2M, and SOD3 correlated with increasing age. We found that SPP2, ADIPOQ, SOD3, APOE, ITIH2, and PKM were expressed in higher levels in women compared to men. To ensure that the age/sex correlations identified in these proteins did not affect the expression related to the differences between PD and control samples, we adjusted the data for age and sex and obtained Benjamini-Hochberg multiple testing corrected p-values for the comparison between PD and HC and related these to the p-values from the unadjusted model. Apart from CST3 and VCAM1, all results remained unchanged. Since PTGDS made part of the predictive SVM panel, we removed this variable from the model to ascertain that the classification was not biased by age. We compared the outcomes from the models with and without PTGDS and found that they produced identical results in the initial test and training dataset, thus indicating that the model is not influenced by age-protein correlations.

Supplementary Methods 3. Evaluation of the larger iRBD cohort, with longitudinal samples (Phase II)

The longitudinal iRBD samples (146, phase II) were applied to (i) the PD vs. control OPLS-DA model that had been constructed using the initial sample set, and (ii) our refined and simplified discriminant support vector machine model consisting of eight proteins. The OPLS-DA model contained the detectable proteins and resulted in 107 of 146 iRBD samples being identified as PD or control, and 39 samples as unclassified. Analysis of these new iRBD samples demonstrated that 70% were consistently identified as having a PD panel profile. Longitudinally, 22 of the 40 individuals with longitudinal follow-up samples were consistently classified as PD. The SVM model classified 79% of the samples as PD. At the time of analysis, 16 of the 54 subjects in our longitudinal iRBD validation cohort had developed PD/DLB. Out of these samples with a known clinical outcome, our SVM model identified ten individuals with all their timepoints as PD. Of the 11 PD converters, eight were identified as PD. The earliest correct classification was 7.3 years prior to diagnosis and the latest was 0.9 years prior to diagnosis (average 3.5 ± 2.4 years). In 26 of the 40 individuals with longitudinal follow-up samples the baseline samples and all longitudinal samples demonstrated a PD profile, while four individuals had all their timepoints classified as controls. Ten individuals demonstrated indeterminate predictions, with timepoints from the same individual showing a PD profile in most, but not all samples. Generally, the samples predicted as controls in the SVM model corresponded to the samples with no prediction outcome in the OPLS-DA model. In those subjects who converted to PD/DLB (stage 3 NSD) during follow-up, eight of eleven were observed to have the PD profile through all timepoints and the remaining three patients demonstrated a change to a PD profile during follow-up. (**Supplementary Figure S6**).

Supplementary Notes 1. Members of the PROPAG-AGING consortium

Astrid Adarmes-Gómez,
Tiago Azevedo,
Maria-Giulia Bacalini,
Luca Baldelli,
Anna Bartoletti-Stella,
Kailash P. Bhatia,
Bonilla-Toribio Marta,
Claudia Boninsegna,
Marcella Broli,
Buiza-Rueda Dolores,
Giovanna Calandra-Buonaura,
Sabina Capellari,
Mario Carrión-Claro,
Rosalia Cilea,
Robert Clayton,
Pietro Cortelli,
Alessandra Dal Molin,
Silvia De Luca,
Patrizia De Massis,
Giovanna Maria Dimitri,
Ivan Doykov,
Rocio Escuela-Martin,
Giovanni Fabbri,

Claudio Franceschi,
Anna Gabellini,
Paolo Garagnani,
Cristina Giuliani,
Pilar Gómez-Garre,
Pietro Guaraldi,
Sara Hägg,
Jenny Hällqvist,
Claire Halsband,
Wendy Heywood,
Henry Houlden,
Ismae Huertas,
Silvia Jesús,
Juulia Jylhävä,
Miguel A. Labrador-Espinosa,
Cristina Licari,
Pietro Liò,
Claudio Luchinat,
Daniel Macias,
Stefania Macrì,
Francesca Magrinelli,
Juan Francisco Martín Rodríguez,
Delledonne Massimo,
Maria Giovanna Maturo,
Giacomo Mengozzi,
Gaia Meoni,
Francesco Mignani,
Maddalena Milazzo,
Kevin Mills,
Pablo Mir,
Brit Mollenhauer,
Christine Nardini,
Stefania Alessandra Nassetti,
Nancy L. Pedersen,
Maria Teresa Periñán-Tocino,
Chiara Pirazzini,
Federica Provini,
Francesco Ravaioli,
Claudia Sala,
Luisa Sambati,
Cesa Lorella Maria Scaglione,
Sebastian Schade,
Sebastian Schreglmann,
Simeon Spasov,
Stephen Strom,
Cristina Tejera-Parrado,
Leonardo Tenori,
Claudia Trenkwalder,
Paola Turano,
Franco Valzania,
Rosario Vigo Ortega,
Dylan Williams,
Luciano Xumerle
& Elisa Zago

Supplementary Tables

Supplementary Table 1. ***Characteristics of the Parkinson's disease subjects and healthy controls included in the discovery proteomics study (phase 0):*** *The table shows age, sex, MDS-UPDRS part III and MMSE score in addition to the disease duration for each individual. Abbreviations: MMSE= Mini-Mental State Examination, UPDRS= Unified Parkinson's Disease Rating Scale, DNP= de novo Parkinson's disease; HC= Healthy control; SD= Standard deviation*

Subject	Age (years)	Sex	MDS-UPDRS III	MMSE	Disease duration (months)
**DNP01**		Male	21	30	18
**DNP02**		Male	20	30	72
**DNP03**		Male	17	29	17
**DNP04**		Male	28	29	24
**DNP05**		Male	6	29	8
**DNP06**		Female	56	28	20
**DNP07**		Male	35	28	9
**DNP08**		Male	15	30	12
**DNP09**		Male	66	28	24
**DNP10**		Female	35	27	20
**Average (± SD)**	**66.1 (10.8)**	**8 Male**	**29.9 (17.8)**	**28.8 (0.98)**	**22.4 (17.4)**
**HC01**		Female	0	30	n/a
**HC02**		Female	0	28	n/a
**HC03**		Female	0	30	n/a
**HC04**		Female	1	29	n/a
**HC05**		Male	2	29	n/a
**HC06**		Male	4	30	n/a
**HC07**		Male	0	27	n/a
**HC08**		Male	0	29	n/a
**HC09**		Female	4	28	n/a
**HC10**		Male	0	29	n/a
**Average (± SD)**	**65.7 (8.6)**	**5 Male**	**1.1 (1.6)**	**28,9 (0.94)**	

Supplementary Table 2. ***Peptides included in the targeted proteomic assay (phases I and II)*****:** *The peptides are represented by gene name, followed by protein name, and peptide amino acid sequence. The transitions from precursor ion to product ions (quantifier and qualifier) are also shown. Internal standards are found at the bottom of the table in italics.*

**Gene**	**Protein**	**AA sequence**	**Transitions (precursor > quant/qual)**
A2M	Alpha-2-macroglobulin	AFQPFFVELTMPYSVIR	1023.03 > 1079.59/1208.63
ADIPOQ	Adiponectin	IFYNQQNHYDGSTGK	591.27 > 666.32/1106.49
ANXA2	Annexin A2	TNQELQEINR	622.82 > 659.35/772.43
		TPAQYDASELK	611.8 > 825.4/1024.49
APOE	Apolipoprotein E	LGPLVEQGR	484.78 > 588.31/701.39
APP	Amyloid-beta precursor protein	GLTTRPGSGLTNIK	472.27 > 572.33/622.85
		YLETPGDENEHAHFQK	638.96 > 767.39/1010.48
ATIC	Bifunctional purine biosynthesis protein PURH	DVSELTGFPEMLGGR	804.39 > 759.38/963.47
		HVSPAGAAVGIPLSEDEAK	616.65 > 847.44/888.43
BCAP29	B-cell receptor-associated protein 29	SSTSRPDAYEHTQMK	435.2 > 468.72/504.23
BCHE	Cholinesterase	YGNPNETQNNSTSWPVFK	1041.98 > 490.3/874.92
		YLTLNTESTR	599.31 > 707.33/921.46
C15orf62	Chromosome 15 Open Reading Frame 62	EQFPSEPSF	534.24 > 350.17/718.3
		LPLWGDEQPR	605.81 > 701.32/887.4
C3	Complement C3	TVMVNIENPEGIPVK	820.44 > 853.48/982.52
CAPN2	Calpain-2 catalytic subunit	NFFLTNR	456.24 > 650.36/797.43
		SDTFINLR	483.26 > 515.33/763.45
CCL2	C-C motif chemokine 2	WVQDSMDHLDK	458.54 > 845.38/960.41
CCL4	C-C motif chemokine 4	NFVVDYYETSSLCSQPAVVFQTK	894.76 > 889.47/1104.56
CCL13	C-C motif chemokine 13	SYVITTSR	463.75 > 577.33/676.4
CCL17	C-C motif chemokine 17	DAIVFVTVQGR	602.84 > 806.45/905.52
CCL22	C-C motif chemokine 22	HFYWTSDSCPRPGVVLLTFR	610.31 > 627.89/649.4
CCL24	C-C motif chemokine 24	GQQFCGDPK	518.73 > 576.22/723.29
CCL26	C-C motif chemokine 26	SYEFTSNSCSQR	733.3 > 838.35/939.39
CD200R1	CD200 Receptor 1	QITQNYSK	491.09 > 370.66/740.45
CD22	B-cell receptor CD22	EVQFFWEK	556.77 > 609.3/756.37
CHI3L1	Chitinase-3-like protein 1	VTIDSSYDIAK	606.31 > 696.36/783.39
COL4A2	Collagen alpha-2(IV) chain	GLDGYQGPDGPR	616.29 > 541.27/726.35
		SVSIGYLLVK	539.83 > 692.43/892.55
COL6A3	Collagen alpha-3(VI) chain	QLGTVQQVISER	679.38 > 859.46/1116.6
CPS1	Carbamoyl-Phosphate Synthase 1	FVHDNYVIR	388.2 > 458.74/779.4
CRP	C reactive protein	APLTKPLK	289.86 > 586.39/699.48
		ESDTSYVSLK	564.77 > 696.39/797.44
CS	Citrate synthase, mitochondrial	ALGVLAQLIWSR	663.92 > 448.06/561.1
CSF1R	Colony Stimulating Factor 1 Receptor	NVLLTNGHVAK	583.34 > 625.34/726.39
		VVEATAFGLGK	546.31 > 764.43/893.47
CST3	Cystatin-C	ALDFAVGEYNK	613.81 > 780.39/927.46
CTHRC1	Collagen Triple Helix Repeat Containing 1	GDASTGWNSVSR	618.78 > 805.4/906.44
CUL5	Cullin-5	YVEQLLTLFNR	698.39 > 763.45/876.53
CYCS	Cytochrome c	GIIWGEDTLMEYLENPK	670 > 763.35/892.39
		TGPNLHGLFGR	390.21 > 505.78/534.29

DCXR	L-xylulose reductase	AVIQVSQIVAR	592.36 > 772.47/900.53
		TQADLDSLVR	559.39 > 474.52/888.86
DKK3	Dickkopf WNT signalling pathway inhibitor 3	EVPDEYEVGSFMEEVR	957.92 > 843.87/1053.5
		SAVEEMEAEEAAAK	732.83 > 949.43/1078.47
EFNA5	Ephrin A5	VENSLEPADDTVHESAEPSR	728 > 755.84/820.36
		VFDVNDK	418.71 > 590.28/737.35
ENDOU	Endonuclease, poly(U) specific	YGSEQEFVDDLK	715.33 > 865.43/1266.58
EPO	Erythropoietin	EVWQGLALLSEAVLR	842.78 > 674.61/1141.94
		TITADTFR	462.74 > 609.3/710.35
FABP5	Fatty acid binding protein 5	ELGVGIALR	464.28 > 529.35/685.44
		TTQFSTLGEK	556.29 > 781.41/909.47
FGA	Fibrinogen alpha chain	AQLVDMK	402.72 > 492.25/605.33
		GLIDEVNQDFTNR	760.87 > 894.41/1237.54
FGF2	Basic fibroblast growth factor 2	LESNNYNTYR	637.29 > 716.34/830.38
FGF21	Fibroblast growth factor 21	EDGTVGGAADQSPESLLQLK	672.34 > 464.28/701.46
		YLYTDDAQQTEAHLEIR	689.33 > 813.89/895.42
GRN	Granulin precursor	AVALSSSVMCPDAR	732.35 > 1022.44/1109.47
		ENATTDLLTK	553.29 > 690.4/791.45
HBE1	Haemoglobin subunit epsilon	EFTPEVQAAWQK	717.36 > 830.45/1056.55
		MNVEEAGGEALGR	666.81 > 730.38/859.43
HELLS	Lymphoid-specific helicase	LISQIQPEVDR	649.36 > 743.37/984.51
HPX	Hemopexin	YYCFQGNQFLR	748.34 > 862.45/1009.52
HSPA1L	Heat shock 70 kDa protein 1-like	VEIIANDQGNR	614.82 > 703.31/774.35
HSPA5	Endoplasmic reticulum chaperone BiP	ITITNDQNR	537.78 > 747.34/860.42
HSPA8	Heat shock cognate 71 kDa protein	DAGTIAGLNVLR	600.34 > 742.46/855.54
HSP90AB1	Heat shock protein HSP 90-beta	SIYYITGESK	580.8 > 634.34/797.4
HSPD1	Heat Shock Protein Family D (Hsp60) Member 1	VTDALNATR	480.76 > 645.37/760.39
ICAM1	Intercellular adhesion molecule 1	ASVSVTAEDEGTQR	725.34 > 834.36/1006.44
		LLGIETPLPK	540.84 > 797.48/854.5
IFNG	Interferon gamma	AIHELIQVMAELSPAAK	911.15 > 386.13/473.19
		IMQSQIVSFYFK	745.89 > 903.5/1031.56
IL1A	Interleukin-1 alpha	ESMVVVATNGK	567.79 > 589.33/688.4
IL1B	Interleukin-1 beta	SLVMSGPYELK	612.32 > 793.41/924.45
IL2	Interleukin-2	DLISNINVIVLELK	791.97 > 813.54/927.59
IL4	Interleukin-4	EANQSTLENFLER	775.88 > 807.4/920.48
IL5	Interleukin-5	TLLIANETLR	572.34 > 703.37/816.46
IL6	Interleukin-6	YILDGISALR	560.82 > 616.38/844.49
IL7	Interleukin-7	LNDLCFLK	511.77 > 680.38/795.41
IL10	Interleukin-10	AMSEFDIFINYIEAYMTMK	1159.71 > 379.34/510.43
		DQLDNLLLK	536.31 > 715.43/828.52
IL12A	Interleukin-12 subunit alpha	TSTVEACLPLELTK	781.41 > 700.4/1173.6
IL12B	Interleukin-12 subunit beta	EDGIWSTDILK	638.82 > 676.39/862.47
IL13	Interleukin-13	ELIEELVNITQNQK	835.95 > 944.52/1057.6
IL15	Interleukin-15	TEANWVNVISDLK	744.89 > 788.45/887.52
IL16	Pro-interleukin-16	DPGVSESPPPGR	597.79 > 739.37/925.47
IRAK4	Interleukin-1 receptor-associated kinase 4	SANILLDEAFTAK	696.87 > 781.37/894.46

ITIH2	Inter-alpha-trypsin inhibitor heavy chain 2	TEVNVLPGAK	514.29 > 698.42/797.49
		VQFELHYQEVK	473.91 > 666.35/803.4
LMO7	LIM domain only protein 7	KPQDQLVIER	409.23 > 417.25/710.38
MAPK12	Mitogen-activated protein kinase 12	VTGTPPAEFVQR	651.35 > 472.25/846.45
MASP2	Mannan binding lectin serine peptidase 2	AGYVLHR	408.23 > 425.26/524.33
		WPEPVFGR	494.26 > 575.33/801.43
MMP3	Matrix metallopeptidase 3	GNQFWAIR	496.26 > 545.32/692.39
		TYFFVEDK	524.75 > 637.32/784.39
MSN	Moesin	EDAVLEYLK	540.28 > 552.3/665.39
MUC5B	Mucin 5B, oligomeric mucus/gel-forming	ATNSTATPSSTLGTTR	783.39 > 919.48/1020.53
NCAM1	Neural cell adhesion molecule 1	DGQLLPSSNYSNIK	768.39 > 1009.49/1122.58
		LEGQMGEDGNSIK	689.32 > 819.38/950.42
NDRG1	N-myc downstream regulated 1	ISGWTQALPDMVVSHLFGK	696.03 > 615.32/787.45
		MADCGGLPQISQPAK	786.88 > 868.49/1038.59
NEFL	Neurofilament light polypeptide	VLEAELLVLR	577.86 > 613.44/813.52
		YEEEVLSR	512.75 > 603.35/732.39
NEFM	Neurofilament medium polypeptide	FVEEIIEETK	618.82 > 732.41/990.5
		SIELESVR	466.76 > 490.26/732.39
NEFH	Neurofilament heavy polypeptide	LEQEHLLEDIAHVR	426.23 > 595.37/710.39
		TSVSSVSASPSR	582.8 > 604.3/877.44
NFATC2	Nuclear Factor Of Activated T Cells 2	YQQQNPAAVLYQR	789.9 > 820.47/917.52
NFKBIZ	NF-kappa-B inhibitor zeta	ASGQAVDDFK	519.25 > 694.34/879.42
NLRP3	NLR Family Pyrin Domain Containing 3	YLEDLEDVDLK	676.34 > 831.45/946.47
OLR1	Oxidised low-density lipoprotein receptor 1	QQAEEASQESENELK	573.93 > 719.36/848.4
PGAM1	Phosphoglycerate mutase 1	AMEAVAAQGK	488.25 > 644.37/773.42
PGK1	Phosphoglycerate kinase 1	VLPGVDALSNI	549.5 > 466.54/885.84
PKM	Pyruvate kinase M	ITLDNAYMEK	599.29 > 755.34/791.39
PLAU	Urokinase-type plasminogen activator	SDALQLGLGK	501.28 > 615.38/728.47
PLD3	Phospholipase D Family Member 3	ALLNVVDNAR	542.81 > 574.29/787.41
		LLISCWGHSEPSMR	558.27 > 667.27/723.82
PPP3CB	Protein Phosphatase 3 Catalytic Subunit Beta	GLTPTGMLPSGVLAGGR	792.43 > 656.86/813.46
PRDX3	Peroxiredoxin 3	GLFIIDPNGVIK	643.38 > 742.41/855.49
PRG4	Proteoglycan 4	GFGGLTGQIVAALSTAK	795.95 > 1058.62/1159.67
PTGDS	Prostaglandin-H2 D-isomerase	AQGFTEDTIVFLPQTDK	955.51 > 363.41/588.56
		TMLLQPAGSLGSYSYR	872.44 > 989.47/1157.56
PTGES2	Prostaglandin E synthase 2	QWADDWLVHLISPNVYR	704.54 > 735.46/848.62
RANGAP1	Ran GTPase-activating protein 1	AFNSSSFNSNTFLTR	564.94 > 637.34/751.38
		VINLNDNTFTEK	704.36 > 968.43/1195.56
SAA1	Serum amyloid A-1 protein	EANYIGSDK	498.74 > 682.34/796.38
		SFFSFLGEAFDGAR	776.22 > 303.33/822.63
SELE	E-selectin	YTHLVAIQNK	396.22 > 573.34/672.4
SERPINA1	Alpha-1-antitrypsin	LSSWVLLMK	538.81 > 603.39/876.5
SERPINA3	Alpha-1-antichymotrypsin	LYGSEAFATDFQDSAAAK	946.44 > 952.44/1053.48
SERPINF2	Alpha-2-antiplasmin	HQMDLVATLSQLGLQELFQAPDLR	908.65 > 500.13/1112.82

		LGNQEPGGQTALK	656.85 > 674.38/771.44
SERPING1	Plasma protease C1 inhibitor	LVLLNAIYLSAK	659.41 > 765.45/992.58
SMC4	Structural Maintenance Of Chromosomes 4	SNNIINETTTR	631.82 > 721.35/834.43
SNAP25	Synaptosomal-associated protein 25	AWGNNQDGVVASQPAR	557.27 > 558.3/629.34
		HMALDMGNEIDTQNR	582.26 > 633.3/746.38
SOD2	Superoxide Dismutase 2	LTAASVGVQGSGWGWLGFNK	1018.02 > 1064.53/1208.58
SOD3	Superoxide Dismutase 3	AGLAASLAGPHSIVGR	492.95 > 582.83/618.35
		VTGVVLFR	445.78 > 534.34/690.43
SPP2	Secreted Phosphoprotein 2	DALSASVVK	445.25 > 503.32/590.35
		VNSQSLSPYLFR	705.87 > 695.39/782.42
TEK	Angiopoietin-1 receptor	IVDLPDHIEVNSGK	512.61 > 548.28/662.33
THY1	Thy-1 Cell Surface Antigen	HVLFGTVGVPEHTYR	428.73 > 401.7/479.74
TLR6	Toll-like receptor 6	DMPSLEILDVSWNSLESGR	1074.49 > 948.79/1035.89
TNF	Tumour necrosis factor (alpha)	ANALLANGVELR	620.85 > 758.42/871.5
		DNQLVVPSEGLYLIYSQVLFK	1213.38 > 570.24/669.35
TNFB	Lymphotoxin-alpha	MHLAHSTLKPAAHLIGDPSK	531.79 > 331.2/619.35
TNNT3	Troponin T3	DLMELQALIDSHFEAR	629.98 > 764.89/830.41
TOLLIP	Toll-interacting protein	GPVYIGELPQDFLR	802.43 > 775.41/1074.56
		LNITVVQAK	493.31 > 645.39/758.48
TRAP1	Heat shock protein 75 kDa, mitochondrial	ELGSSVALYSR	591.31 > 609.34/882.47
TUBA4A	Tubulin alpha-4A chain	AVFVDLEPTVIDEIR	858.46 > 942.53/1299.68
		DVNAAIAAIK	493.29 > 586.39/771.47
TXN	Thioredoxin	LEATINELV	501.22 > 474.11/528.24
UBC	Polyubiquitin-C	TITLEVEPSDTIENVK	894.47 > 905.46/1002.51
VCAM1	Vascular cell adhesion protein 1	LHIDEMDSVPTVR	756.59 > 251.23/472.42
		NTVISVNPSTK	580.32 > 732.39/944.54
VEGFA	Vascular endothelial growth factor A	SWSVYVGAR	512.76 > 565.31/751.41
VEGFC	Vascular endothelial growth factor C	FAAAHYNTEILK	459.91 > 580.31/615.83
VEGFD	Vascular endothelial growth factor D	FAATFYDIETLK	709.86 > 718.4/881.46
#N/A	*AP_ALDOA_ALQ*	*ALQASALK*	*404.75 > 496.32/624.38*
#N/A	*AP_C3_SSL*	*SSLSVPYVIVPLK*	*704.43 > 357.25/934.6*
#N/A	*AP_GSTO1_GSA*	*GSAPPGPVPEGSIR*	*664.36 > 556.81/1015.56*
#N/A	*AP_RSU1_ALY*	*ALYLSDNDFEILPPDIGK*	*1014.03 > 633.37/746.37*
#N/A	*AP_TSP1_TIV*	*TIVTTLQDSIR*	*627.36 > 940.52/1039.59*
#N/A	*ENO1_YEAST_GNP*	*GNPTVEVELTTEK*	*709.06 > 623.49/948.68*

Supplementary Table 3. ***Characteristics of longitudinal iRBD subjects (phase II)***
The table shows age, sex, number of longitudinal samples, and the time since baseline for the last sample. Out of the 40 iRBD subjects (27 male), 16 had converted to stage 3 NSD (neuronal synuclein disease) at the time of the last sample (11 Parkinson's disease, five dementia with Lewy bodies). The time from baseline to conversion is shown.

Subject	Age at baseline (years)	Number of longitudinal Follow-up samples	Phenoconversion	Diagnosis
**iRBD01**	65	5	8 years after BL	PD
**iRBD02**	52	5	6 years after BL	PD
**iRBD03**	77	5	8 years after BL	PD
**iRBD04**	64	4		
**iRBD05**	66	4	10 years after BL	DLB
**iRBD06**	71	3		
**iRBD07**	71	5	10 years after Bl	PD
**iRBD08**	62	4		
**iRBD09**	64	5	10 years after Bl	PD
**iRBD10**	73	5		
**iRBD11**	69	5	9 years after Bl	DLB
**iRBD12**	75	4		
**iRBD13**	73	4	9 years after Bl	PD
**iRBD14**	68	3		
**iRBD15**	62	4		
**iRBD16**	64	3	9 years after BL	DLB
**iRBD17**	51	4		
**iRBD18**	63	2		
**iRBD19**	54	3		
**iRBD20**	50	3		
**iRBD21**	77	3	8 years after BL	PD
**iRBD22**	68	4	11 months after BL	DLB
**iRBD23**	73	2		
**iRBD24**	55	3		
**iRBD25**	53	3		
**iRBD26**	68	3	4 years after BL	DLB
**iRBD27**	72	3		
**iRBD28**	69	2	2 years after BL	PD
**iRBD29**	73	2	1 year after BL	PD
**iRBD30**	77	3		
**iRBD31**	68	2		
**iRBD32**	67	3	7 mos after BL	PD
**iRBD33**	74	3		
**iRBD34**	80	3		
**iRBD35**	58	2	1 year after BL	PD
**iRBD36**	76	2		
**iRBD37**	79	2		
**iRBD38**	70	2		
**iRBD39**	74	2		
**iRBD40**	76	2		
**Average ± SD**	**67.5 (8.1)**	**3.3 (1.1)**	**3.7 (2.6)**	

Abbreviations: iRBD= isolated REM sleep behaviour disorder; PD= Parkinson's disease; DLB= Dementia with Lewy bodies; BL= Baseline; SD= Standard deviation

Supplementary Table 4 ***Significant relationships with longitudinal progression evaluated by linear mixed effects models (phase II):*** *The table shows the two-sided Student's t-p-values of the interaction between the time since baseline and each significant clinical variable post Benjamini-Hochberg multiple testing correction (alpha = 0.05), the coefficient and the 95% confidence interval ± the standard error.*

Clinical variable	Significance	Coefficient	95 CI ± SE
Hoehn-Yahr	2.1E-2	0.011	[0.0038, 0.0181] ± 0.0036
UPDRS I	4.6E-2	0.018	[0.0041, 0.0311] ± 0.0069
UPDRS III	3.9E-2	0.098	[0.0252, 0.1698] ± 0.0369
UPDRS I-III	2.1E-2	0.16	[0.0547, 0.2608] ± 0.0526
UPDRS (sum)	2.1E-2	0.16	[0.0549, 0.261] ± 0.0526
PD non-motor symptoms measurement (sum)	1.6E-2	0.12	[0.0938, 0.1474] ± 0.0137
PD non-motor symptoms measurement (mean)	2.1E-2	0.0035	[0.0018, 0.0053] ± 0.0009
Cholesterol	1.6E-2	-0.30	[-0.4841, -0.1186] ± 0.0932

Abbreviations: MMSE= Mini-Mental State Examination, UPDRS= Unified Parkinson's Disease Rating Scale

Supplementary Table 5. ***Significant correlations between cholesterol and proteins measured by targeted mass spectrometry, evaluated by Spearman's correlation (phase II)*****:** *The table shows the two-sided Student's t-test p-values of the correlations between cholesterol and the significant proteins post Benjamini-Hochberg multiple testing correction (alpha = 0.05). The correlation coefficient is also shown. The proteins are annotated by gene name.*

Protein	Significance	Correlation coefficient
HSPA8	4.9E-5	0.50
APOE	2.7E-4	0.45
MASP2	3.4E-3	0.39
PRG4	1.1E-2	0.35
BCHE	1.2E-2	0.31
SERPINA1	3.2E-2	0.31

Supplementary Figures

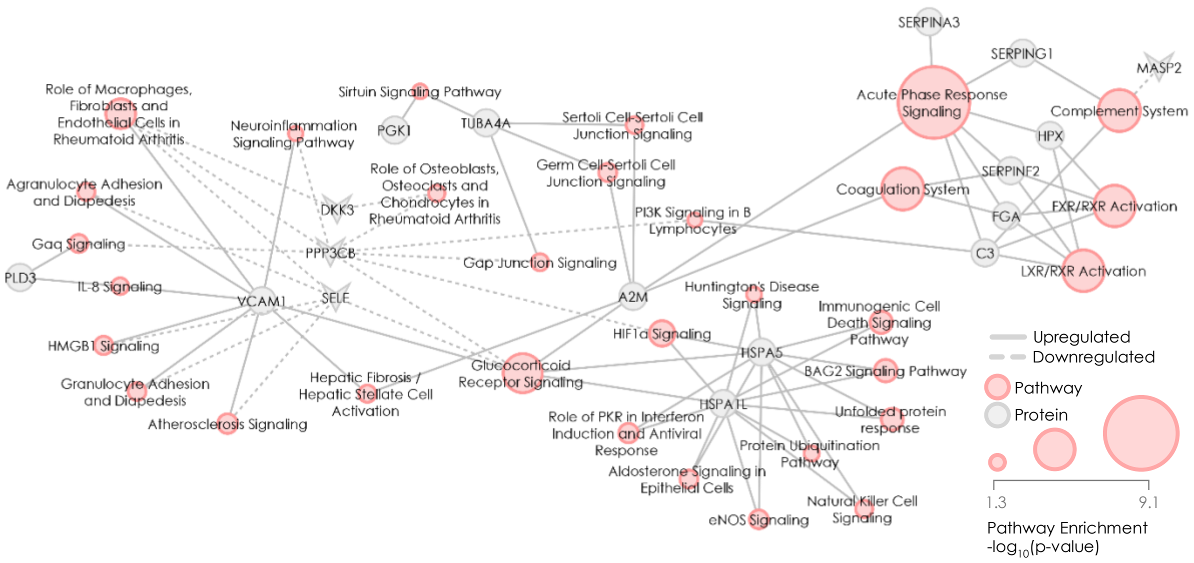

Supplementary Figure 1. ***Pathway enrichment based on the expression of the proteins that were significantly different between PD (n = 99) and controls (n = 36) from the targeted proteomic assay (phase I)***

The analysis was performed in Ingenuity Pathway Analysis using the expression fold-change between the groups. Pathways with an enrichment p-value < 0.05 and involvement of at least two proteins were considered. The pathways are represented by pink nodes and the proteins by grey nodes. Solid edges represent upregulation of a protein in the Parkinson's disease group compared to controls, while dotted edges represent downregulation. The pathways' node sizes represent the significance of the enrichment p-values, larger being more significant. The proteins are annotated by gene names. Source data are provided as a Source Data file.

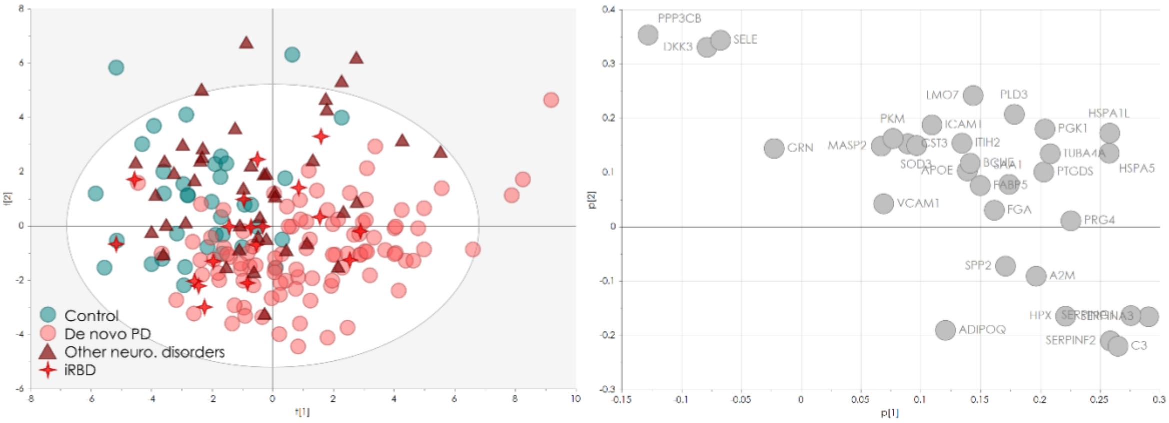

Supplementary Figure 2. ***Principal component analysis of the targeted proteomics data showing the groups de novo PD (DNP), isolated REM sleep behaviour disorder (iRBD), other neurological diseases (OND) and controls (phase I)***

(A) PC1 (23.5%) and PC2 (13.9%) show a separation between de novo PD (n = 99) and controls (n = 36). The iRBD samples (n = 18) distribute between de novo PD and controls while other neurological disorders (n = 41) mainly cluster with controls, indicating that some iRBD patients could be developing the early hallmarks of motor disease (B) The loadings, where the proteins are represented by gene names, demonstrate that de novo PD are correlated with lower levels of PPP3CB, DKK3, SELE, and GRN. Source data are provided as a Source Data file.

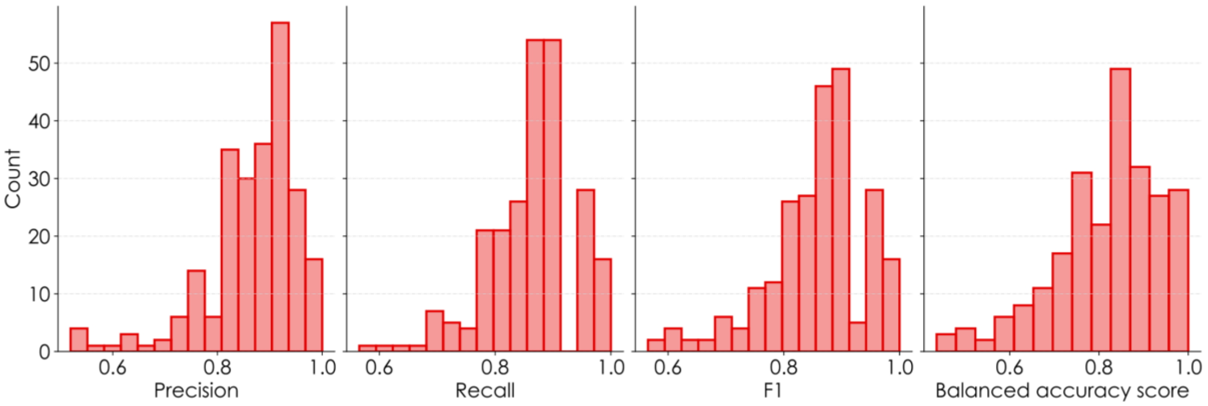

Supplementary Figure 3. ***Classification metrics of the discriminant SVM model, predicting samples as PD or control (phase I)***

The classification metrics were calculated from stratified k-fold cross validation utilising six splits of the data and 40 repetitions, and are displayed as histograms showing the frequency of the metrics precision, recall, the F1 score, and the balanced accuracy score. The average and standard deviation was, for precision 0.87 ± 0.09, for recall 0.87 ±0.08, for the F1 score 0.86 ± 0.09, and for the balanced accuracy score 0.82 ± 0.12. Source data are provided as a Source Data file.

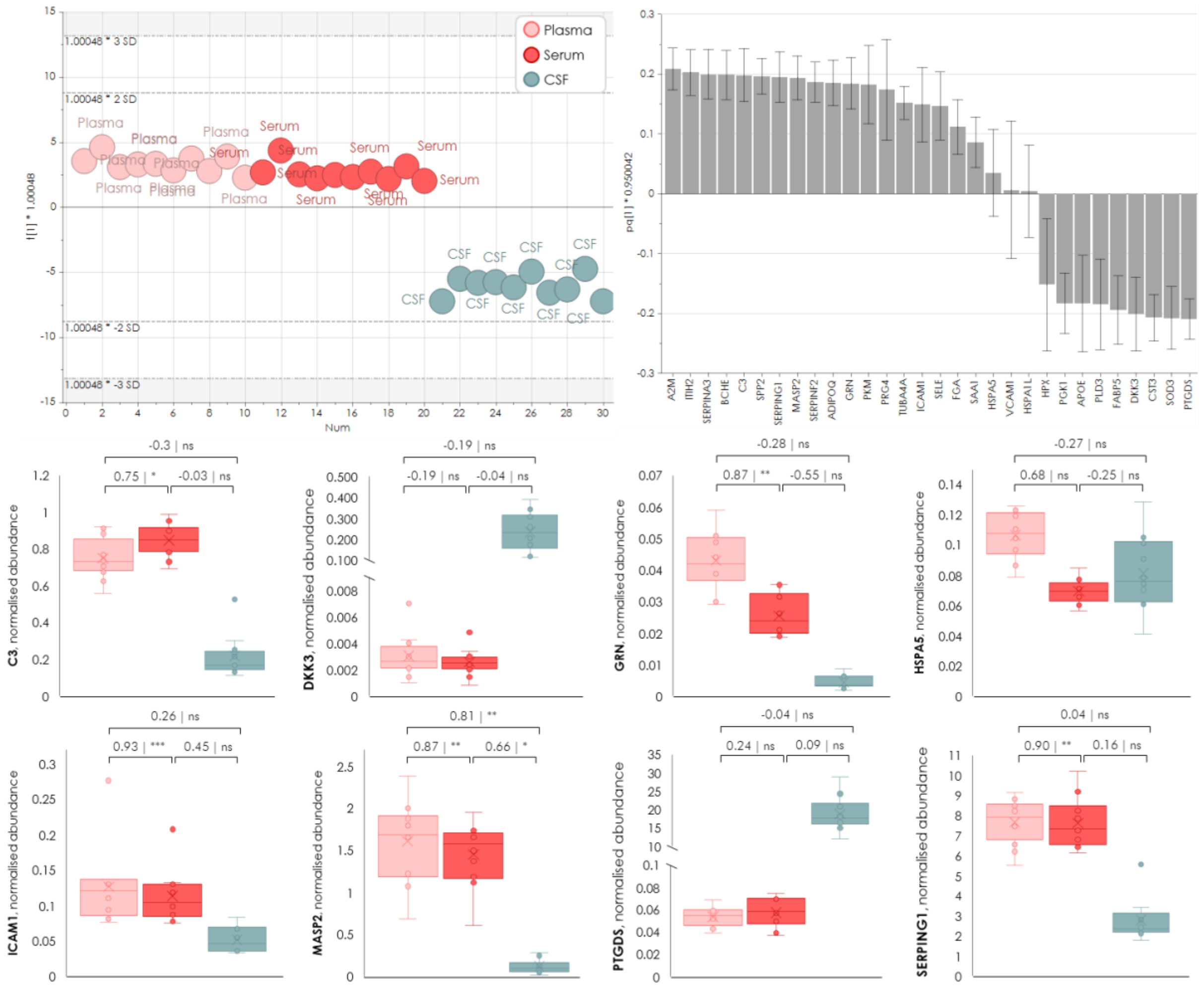

Supplementary Figure 4. ***Protein expression in plasma, serum, and CSF***

*OPLS-DA scores (top left) from a model of plasma (n = 10) and serum (n = 10) versus CSF (n = 10). The model was highly significant with ANOVA p = 2.0E-20 and permutations p << 0.001. The corresponding loadings (top right), where the proteins are represented by gene names, demonstrated that all but three proteins were significantly different between the blood-based plasma and serum, and CSF. Most of the proteins were elevated in plasma/serum, though HPX, PGK1, APOE, PLD3, FABP5, DKK3, CST3, SOD3, and PTGDS were higher in CSF. Box and Whisker plots of the paired plasma, serum, and CSF samples, annotated with Spearman's rho and Benjamini-Hochberg multiple testing adjusted (alpha = 0.05) p-value significance levels from Student's two-sided t-test. The correlation demonstrated that five out of eight of the proteins included in the predictive SVM model were significantly correlated between plasma and serum, . Only MASP2 exhibited significant correlations between CSF and plasma/serum. The whiskers show the minimum and maximum and the boxes show the 25th percentile, the median and the 75th percentile. *** p < 0.001, ** p < 0.01, * p < 0.05, and ns = not significant. Source data are provided as a Source Data file.*
