# Supplementary material for: Plasma proteomics identify biomarkers predicting Parkinson’s disease up to 7 years before symptom onset
Source: Nat Commun. 2024 Jun 18;15:4759. doi: 10.1038/s41467-024-48961-3 (PMC11189460; doi:10.1038/s41467-024-48961-3)
Supplement: Supplementary file 3 — Reporting Summary [file 41467_2024_48961_MOESM3_ESM.pdf]

nature portfolio

Corresponding author(s): Jenny Hällqvist, Michael Bartl

Last updated by author(s): May 15, 2024

Reporting Summary

Nature Portfolio wishes to improve the reproducibility of the work that we publish. This form provides structure for consistency and transparency in reporting. For further information on Nature Portfolio policies, see our Editorial Policies and the Editorial Policy Checklist.

Statistics

For all statistical analyses, confirm that the following items are present in the figure legend, table legend, main text, or Methods section.

n/a	Confirmed	
☐	☒	The exact sample size (*n*) for each experimental group/condition, given as a discrete number and unit of measurement
☐	☒	A statement on whether measurements were taken from distinct samples or whether the same sample was measured repeatedly
☐	☒	The statistical test(s) used AND whether they are one- or two-sided<br>*Only common tests should be described solely by name; describe more complex techniques in the Methods section.*
☐	☒	A description of all covariates tested
☐	☒	A description of any assumptions or corrections, such as tests of normality and adjustment for multiple comparisons
☐	☒	A full description of the statistical parameters including central tendency (e.g. means) or other basic estimates (e.g. regression coefficient) AND variation (e.g. standard deviation) or associated estimates of uncertainty (e.g. confidence intervals)
☐	☒	For null hypothesis testing, the test statistic (e.g. *F*, *t*, *r*) with confidence intervals, effect sizes, degrees of freedom and *P* value noted<br>*Give P values as exact values whenever suitable.*
☐	☒	For Bayesian analysis, information on the choice of priors and Markov chain Monte Carlo settings
☒	☐	For hierarchical and complex designs, identification of the appropriate level for tests and full reporting of outcomes
☐	☒	Estimates of effect sizes (e.g. Cohen's *d*, Pearson's *r*), indicating how they were calculated

Our web collection on statistics for biologists contains articles on many of the points above.

Software and code

Policy information about availability of computer code

Data collection	Targeted mass spectrometric data were collected by the vendor software MassLynx (version 4.1). Peak picking and integrations were performed in TargetLynx (part of the MassLynx suite), or using an in-house application written in Python which can be found at GitHub (https://github.comAchallqvist/mrmIntegrate).
Data analysis	SIMCA, version 17 (Umetrics Sartorius Stedim, Umeå, Sweden) was utilised for multivariate analyses. Most of the statistical analyses were performed in Python (version 3.8.5), applying the packages Statsmodels (version 0.14.0), SciPy (version 1.9.3), SciKit Learn (version 1.1.2), and pymer4 (version 0.8.0). Plots of the data were constructed using the Seaborn and Matplotlib packages (versions 0.12.2 and 3.6.0, respectively).<br><br>The code used for analyses is available via GitHub (https://github.com/jchallqvist/DNP_Pub/blob/main/DNP_Code, doi: https://zenodo.org/doi/10.5281/zenodo.11130369).<br><br>Data were analysed for pathway enrichment using IPA (QIAGEN Inc., https://www.qiagenbioinformatics.com/products/ingenuitypathway-analysis). Gene Ontology (GO) annotations were extracted using DAVID Bioinformatics Resources (2021 build). Networks were built in Cytoscape (version 3.8.0) by applying the “Organic layout” from yFiles.

For manuscripts utilizing custom algorithms or software that are central to the research but not yet described in published literature, software must be made available to editors and reviewers. We strongly encourage code deposition in a community repository (e.g. GitHub). See the Nature Portfolio guidelines for submitting code & software for further information.

Data

Policy information about availability of data

All manuscripts must include a data availability statement. This statement should provide the following information, where applicable:

- Accession codes, unique identifiers, or web links for publicly available datasets
- A description of any restrictions on data availability
- For clinical datasets or third party data, please ensure that the statement adheres to our policy

Peak-picking and integrations were performed in TargetLynx (part of the MassLynx suite, version 4.1), or using an in-house application written in Python which can be found on GitHub (https://github.com/jchallqvist/mrmIntegrate). The raw targeted chromatograms are available to view and download via the Panorama repository (https://panoramaweb.org/DNP_Pub.url). Source data are provided as a Source Data file.

Research involving human participants, their data, or biological material

Policy information about studies with human participants or human data. See also policy information about sex, gender (identity/presentation), and sexual orientation and race, ethnicity and racism.

Reporting on sex and gender	We evaluated the influence of sex in our analyses by comparing sex-adjusted data to non-adjusted data. We adjusted the data for age and sex and calculated the significance in the difference between the disease groups and controls, and compared these results to the significances achieved from the non-adjusted data. We found that two proteins differed in this comparison, though neither of them were included in out predictive machine learning model.
Reporting on race, ethnicity, or other socially relevant groupings	Our study group is composed of subjects with Parkinson's disease, healthy controls and REM sleep disturbance behavior disorder. They were recruited at the Paracelsus Elena-Clinic, Kassel, Germany. We did not collect data regarding ethnicity. We included all the subjects that fullfilled the reported inclusion criteria without a social subgrouping. We did not collect social grouping data.
Population characteristics	Our initial sample set included in the targeted proteomic analysis consisted of plasma from 99 patients newly diagnosed with Parkinson's disease (PD), 67.1 +/- 10.6 years, 36 healthy controls, 63.7 +/- 6.5 years, 41 patients with other neurological disorders (OND), 70 +/- 8.9 years, and 18 patients with REM sleep disturbance behavior disorder (iRBD) 67.3 +/- 8.3 years. The PD and iRBD group did not differ in age compared to the controls, but the OND group was significantly older. In the additional set of iRBD samples, serum from 54 individuals was included. Out of these, 20 had longitudinal follow up samples, rendering a total of 146 samples. The age at baseline for these new samples was 67.5 +/- 8.1 years, not differing significantly from the initial control and iRBD groups. The samples were characterized by several clinical rating scales relevant to Parkinson's disease, such as MMSE and UPDRS. These ratings were evaluated for correlations with the expression of the proteins and are presented in Figure 4, and Supplementary Tables S3 and S6.
Recruitment	During the recruitment period, we specifically asked the referring neurologists to send de novo PD subjects for a thorough clinical evaluation in our inpatient hospital. Healthy controls were recruited through relatives and friends of the enrolled subjects and other patients of our clinic as well as through a newspaper advertisement in early 2009. We offered a free health check for all controls. Screening was first performed by a neurologist specializing in movement disorders. Subjects meeting the inclusion criteria were evaluated by an independent second movement disorder specialist and received a standardized program of investigations.<br>Subjects had to be between 40 and 85 years old, newly diagnosed with PD featuring at least 2 of resting tremor, bradykinesia, and rigidity according to UKBBC and had to fulfill de novo criteria with L-dopa exposure no longer than 2 weeks and not within 4 weeks prior to study entry. Exclusion cirteria whre 1) known severe vascular encephalopathy or normal-pressure hydrocephalus (NPH) as shown on MRI when available at screening or when detected during imaging studies or 2) signs or symptoms according to multiple system atrophy or progressive supranuclear palsy (according to consensus criteria), or medication-induced PD. Healthy controls had to be between 40 and 85 years old, without any active known/treated condition of the CNS and without a family history of idiopathic PD. Antipsychotic drugs were an exclusion criterion both for controls and patients. Controls were matched using frequency matching by age, sex, and education.
Ethics oversight	Institutional review board statements were obtained from the University Medical Centre in Goettingen, Germany Approval No. 9/7/04 and 36/7/02. The study was conducted according to the Declaration of Helsinki and all participants gave written informed consent.

Note that full information on the approval of the study protocol must also be provided in the manuscript.

Field-specific reporting

Please select the one below that is the best fit for your research. If you are not sure, read the appropriate sections before making your selection.

☒ Life sciences ☐ Behavioural & social sciences ☐ Ecological, evolutionary & environmental sciences

For a reference copy of the document with all sections, see nature.com/documents/nr-reporting-summary-flat.pdf

Life sciences study design

All studies must disclose on these points even when the disclosure is negative.

Sample size	In the proteomic discovery study we performed deep phenotyping of the samples which included considerable on-line fractionation time (> 12 hours per sample). We therefore restricted the size to a limited number of samples (n = 20) as to not introduce irreparable instrumental drift. In the targeted analyses, we relied on replication and cross-validation to verify our results and did not perform any prior sample size calculations. We developed a validatory targeted assay to confirm the discovery findings, containing targets from the discovery study and from pathways implicated by the discovery study. In the initial targeted validation proteomics study of plasma, we included a total of 194 samples, consisting of 99 PD and 36 controls (theoretically sufficient for a 75% power detectable 1.5 fold change (Levin, Y. (2011), The role of statistical power analysis in quantitative proteomics. Proteomics, 11: 2565-2567. https://doi.org/10.1002/pmic.201100033), we further included 18 iRBD samples to evaluate if there was a prodromal expression, and 41 OND as positive controls. We verified the prodromal iRBD expression identified in the initial targeted analysis by predicting 146 new iRBD samples in the machine learning model.
Data exclusions	Extreme outliers, data points deviating more than ten median absolute deviations, were excluded.
Replication	The initial targeted proteomic analysis resulted in predictive machine learning models, where the support vector machine model proved capable of discriminating between PD and controls with 100% accuracy, and additionally predicting the proportion of iRBD samples projected as PD on a scale corresponding to the theoretical clinical conversion rate of iRBD to a synucleopathy (72% in the OPLS-DA model and 94% in the support vector machine model (SVM)). We evaluated if these results could be replicated by analysing a new set of longitudinal iRBD samples (n = 146) which were predicted in the SVM model and resulted in 79% of the iRBD samples predicted as PD.
Randomization	For instrumental analysis, the samples were randomised by the "constrained randomisation" strategy, thus randomising within and between blocks of samples consisting of all conditions.
Blinding	The investigators were blinded to group allocation during data collection.The investigators preparing the samples for analysis were blinded. The investigators performing the instrumental analysis were unblinded to be able to construct the constrained randomisation described above.

Reporting for specific materials, systems and methods

We require information from authors about some types of materials, experimental systems and methods used in many studies. Here, indicate whether each material, system or method listed is relevant to your study. If you are not sure if a list item applies to your research, read the appropriate section before selecting a response.

Materials & experimental systems

n/a	Involved in the study	
☒	☐	Antibodies
☒	☐	Eukaryotic cell lines
☒	☐	Palaeontology and archaeology
☒	☐	Animals and other organisms
☐	☒	Clinical data
☒	☐	Dual use research of concern
☒	☐	Plants

Methods

n/a	Involved in the study	
☒	☐	ChIP-seq
☒	☐	Flow cytometry
☒	☐	MRI-based neuroimaging

Clinical data

Policy information about clinical studies
All manuscripts should comply with the ICMJEguidelines for publication of clinical research and a completedCONSORT checklist must be included with all submissions.

Clinical trial registration	Institutional review board statements were obtained from the University Medical Centre in Goettingen, Germany Approval No. 9/7/04 and 36/7/02. The study was conducted according to the Declaration of Helsinki and all participants gave written informed consent.
Study protocol	Additional information can be found here: https://drks.de/search/de/trial/DRKS00000540
Data collection	Biannual longitudinal data were collected at baseline (BL) and every 24-months over 10 years. This includes extensive clinical investigations andre-evaluation of the diagnosis as well as sample collections including CSF, serum and plasma.
Outcomes	DeNoPa is a non-interventional diagnostic study. One outcome measure is the convertion from iRBD to higher stages of neuronal synuclein disease.

Plants

Seed stocks	*Report on the source of all seed stocks or other plant material used. If applicable, state the seed stock centre and catalogue number. If plant specimens were collected from the field, describe the collection location, date and sampling procedures.*
Novel plant genotypes	*Describe the methods by which all novel plant genotypes were produced. This includes those generated by transgenic approaches, gene editing, chemical/radiation-based mutagenesis and hybridization. For transgenic lines, describe the transformation method, the number of independent lines analyzed and the generation upon which experiments were performed. For gene-edited lines, describe the editor used, the endogenous sequence targeted for editing, the targeting guide RNA sequence (if applicable) and how the editor was applied.*
Authentication	*Describe any authentication procedures for each seed stock used or novel genotype generated. Describe any experiments used to assess the effect of a mutation and, where applicable, how potential secondary effects (e.g. second site T-DNA insertions, mosiacism, off-target gene editing) were examined.*
